# Antibiotic and Desiccation Resistance of *Cronobacter sakazakii* and *C. malonaticus* Isolates from Powdered Infant Formula and Processing Environments

**DOI:** 10.3389/fmicb.2017.00316

**Published:** 2017-03-02

**Authors:** Peng Fei, Yujun Jiang, Jing Feng, Stephen J. Forsythe, Ran Li, Yanhong Zhou, Chaoxin Man

**Affiliations:** ^1^Key Laboratory of Dairy Science, Ministry of Education, College of Food Science, Northeast Agricultural UniversityHarbin, China; ^2^National Research Center of Dairy Engineering and Technology, Northeast Agricultural UniversityHarbin, China; ^3^Pathogen Research Group, School of Science and Technology, Nottingham Trent UniversityNottingham, UK

**Keywords:** *C. sakazakii*, *C. malonaticus*, desiccation, antibiotic susceptibility, powdered infant formula

## Abstract

This study evaluated the antimicrobial and desiccation resistance of *Cronobacter sakazakii* and *Cronobacter malonaticus* isolates from powdered infant formula and processing environments. The antimicrobial susceptibility tests showed that the 70 *Cronobacter* strains, representing 19 sequence types, were susceptible to the most of the antibiotics except for amoxicillin-clavulanate, ampicillin, and cefazolin. Furthermore, the growth of six *C. sakazakii* and two *C. malonaticus* strains from different sequence types (STs) in hyperosmotic media was measured. The growth of the two *C. sakazakii* strains (CE1 and CE13) from the neonatal pathovars ST4 and ST8, were significantly higher (*p* < 0.05) than that of other strains. *C. malonaticus* strain CM35 (ST201) was the slowest grower in all strains, and most could not grow in more than 8% NaCl solution. Also the survival of these strains under desiccation conditions was followed for 1 year. The viable count of *Cronobacter* spp. under desiccation conditions was reduced on average by 3.02 log cycles during 1 year, with CE13 (ST8) being the most desiccation resistant strain. These results will improve our understanding of the persistence of the two closely related species *C. sakazakii* and *C. malonaticus* which are of concern for neonatal and adult health.

## Introduction

*Cronobacter* spp. (formerly *Enterobacter sakazakii*) is an emerging opportunistic bacterial pathogen comprised of seven species: *Cronobacter sakazakii, Cronobacter malonaticus, Cronobacter turicensis, Cronobacter muytjensii, Cronobacter condimenti, Cronobacter universalis*, and *Cronobacter dublinensis* (Joseph et al., 2012b; Lu et al., 2014). This organism can cause necrotizing enterocolitis, bacteremia, and meningitis in neonates and infants, with a 40–80% mortality rate (Forsythe et al., 2014; Holy and Forsythe, 2014; Li et al., 2015). To date, only the three species *C. sakazakii, C. malonaticus*, and *C. turicensis* have been isolated from neonatal infections (Sonbol et al., 2013). *Cronobacter* spp. has been recovered from a wide variety of foods, with powdered infant formula (PIF) being of particular concern as it is the most significant source of *Cronobacter* strains resulting in neonatal infections (Iversen and Forsythe, 2004; Craven et al., 2010). *Cronobacter* strains cannot survive after the standard pasteurization procedures, therefore, the addition of non-heat treated materials and environmental contamination during filling and packaging are the plausible causes of *Cronobacter* contamination of PIF (Nazarowec-White and Farber, 1997). Besides, the strong resistant ability of the organism in dry PIF factory environments can increase the likelihood of post-pasteurization contamination (Riedel and Lehner, 2007).

Currently, antibiotic therapy is considered to be the common and preferred way to prevent the *Cronobacter* infection in humans (Depardieu et al., 2007). Many studies have confirmed that *Cronobacter* strains can be effectively eliminated by antibiotics, however, prolonged use of antibiotics is undesirable as it may result in the development of *Cronobacter* antibiotic resistance (Yoneyama and Katsumata, 2006; McMahon et al., 2007). Hochel et al. (2012) reported that all of 53 *Cronobacter* strains isolated from 399 retail food samples were resistant to erythromycin, and two of them were resistant to both erythromycin and tetracycline. Chon et al. (2012) found that 77.8% *Cronobacter* strains isolated from desiccated foods in Korea were not susceptible to cephalothin. The *Cronobacter* strains resistance to cefotaxime and streptomycin have also been isolated from various foods in Korea and commercial PIF in China (Lee et al., 2012). Caubilla-Barron and co-workers reported that the *C. sakazakii* strains isolated from two fatal neonatal infections expressed β–lactamase activity (Caubilla-Barron et al., 2007). In addition, the effective dose of antibiotics for treatment may change after long-term antibiotic use, therefore screening environmental and PIF isolates of *Cronobacter* for antibiotic resistance would be a useful comparison with clinical isolates.

The greater resistance of *Cronobacter* spp. to desiccation compared with other *Enterobacteriaceae* will enable their long term survival under a low water activity (*a*_w_) condition such as in PIF (*a*_w_: 0.2–0.5) which has a long shelf life of up to 1 year (Gurtler and Beuchat, 2007). In general, the viability of *Cronobacter* spp. in PIF decreases by 0.5–0.6 log cycles per month (Edelson-Mammel et al., 2005; Gurtler and Beuchat, 2007). Caubilla-Barron et al. (2007) found there was considerable reduction in the viable count of *Cronobacter* strains (about 3.34 log_10_ cycles) in the first 6 months, whereas after the following 24 months, the average reduction in viability decreased by 1.88 log_10_ cycles. It is important to note that some capsulated *C. sakazakii* strains were still recovered after 2.5 years, by contrast, both *Salmonella enteritidis*, and *Escherichia coli* were undetectable after 15 months (Barron and Forsythe, 2007). Some difference in desiccation resistance can be found among the different *Cronobacter* strains, for example, *C. sakazakii* NCTC11467^T^ has atypical growth characteristics compared with most of *Cronobacter* strains, and cannot be recovered from desiccated condition after 1 year (Iversen et al., 2004). *Cronobacter* spp. produce highly mucoid colonies on milk agar plates and the capsular material could be linked to virulence traits such as macrophage survival as well as desiccation resistance (Ogrodzki and Forsythe, 2015). Particular capsule profiles correlate with the meningitic *C. sakazakii* pathovar clonal complex (CC) 4 (Ogrodzki and Forsythe, 2015).

*Cronobacter* is a diverse genus and has been extensively studied using multilocus sequence typing for over 1,000 strains (Forsythe et al., 2014). This has revealed the high clonality within the genus as well as identified particular pathovars. *C. sakazakii* sequence type (ST) four identified using multi-locus sequence typing (MLST) was the dominant ST, and was associated with neonatal meningitis (Joseph and Forsythe, 2011). In addition, *Cronobacter* pathovars, associated with clonal complex are now recognized. Of particular relevance is the *C. sakazakii* CC4 pathovar which is associated with neonatal meningitis (Hariri et al., 2013; Forsythe et al., 2014). *C. sakazakii* ST8 and *C. sakazakii* ST1 were mainly isolated from clinical sources and PIF, respectively (Forsythe et al. 2014). *C. sakazakii* CC4 was also the genotype of ~25% of isolates recovered from the processing environment of PIF manufacturing plants (Sonbol et al., 2013). Why this CC predominates in PIF and neonatal infections is uncertain since no particular virulence genes have been detected (Joseph et al., 2012a). Therefore, a better understanding of the environmental fitness and antibiotic resistance of *Cronobacter* isolates with significant sequence type from PIF production areas is warranted.

In previous studies, our group isolated 66 *Cronobacter sakazakii* and four *C. malonaticus* strains from PIF and processing environments from 2009 to 2012, and identified their sequence type by MLST (Fei et al., 2015). In this study, we further characterized the strains according to their antibiograms and desiccation tolerance, and considered whether the differences corresponded with their sequence type.

## Materials and methods

### Bacterial strains

A total of 70 *Cronobacter* strains (66 *C. sakazakii* and four *C. malonaticus* strains) were studied. All strains had been isolated from PIF and processing environments, as previously reported (Fei et al., 2015). All 70 *Cronobacter* strains were used for the antimicrobial assays, and six *C. sakazakii* (CE21, CE1, CE13, CE38, CE52, and CE25) and two *C. malonaticus* (CM3 and CM35) strains with different sequence types were selected for studying the survivals under desiccation condition.

Strains were recovered from storage at −80°C in 40% (v/v) glycerol, by inoculating 0.1 mL portions of thawed cultures into 10 mL Luria-Bertani (LB) broth, followed by cultivation at 37°C for 12 h. The cultures were streaked onto Tryptic Soy Agar (TSA) plates and incubated at 37°C for 24 h for single colony isolation. A single colony of each strain was inoculated into the LB and incubated at 37°C for 18 h for following study.

### Antimicrobial susceptibility testing

The antimicrobial susceptibility determination was performed using the BD PhoenixTM^100^ Automated Microbiology System (BD Diagnostic Systems, Sparks, MD) as according to the manufacturer's instructions. Twenty-one antibiotics were selected for the susceptibility test, including amikacin, amoxicillin-clavulanate, ampicillin, ampicillin-sulbactam, aztreonam, cefotaxime, ceftazidime, cefazolin, cefepime, chloramphenicol, ciprofloxacin, colistin, gentamicin, imipenem, levofloxacin, meropenem, moxifloxacin, piperacillin, piperacillin-tazobactam, tetracycline, and trimethoprim-sulfamethoxazole. The results were expressed as sensitive (S), intermediate (I), and resistant (R), and resistant according to the PhoenixTM^100^ guidelines. *E. coli* ATCC 25922 and *E. coli* ATCC 35218 were used as the quality control organisms, and were included in each run according to the manufacturer's recommendations.

### Resistance to osmotic stress

For determining bacterial growth in a hyperosmotic environment, cultures (0.1 mL) were inoculated into brain–heart infusion (BHI) broth containing molar equivalents of either sodium chloride (NaCl) (4, 6, 8, 10% w/v) or sorbitol (12.5, 19, 25, 31% w/v). The above cultures were incubated at 37°C for 24 h, and subsequent growth was measured the growth according to the absorption values at 600 nm (OD 600) using an ultraviolet spectrophotometer (Biochrom Ltd., Cambridge, England). The “time to detection” (TTD), defined as the time (in h) to reach an OD 600 of 0.2, and “growth rate” (in h^−1^) were used to determine the growth of each strain under the different treatments, as previously reported (Alvarez-Ordonez et al., 2014).

### Resistance to dry stress

*C. sakazakii* and *C. malonaticus* were grown in LB for 18 h at 37°C before analysis. For dry stresses, the cultures were harvested by centrifugation at 8,000 g for 10 min, then the cell pellets were diluted in 0.85% sterile normal saline (NS) to obtain a cell density of 8.00 log_10_ cfu/mL.

The *C. sakazakii* and *C. malonaticus* cell suspensions were desiccated as previously described with minor modifications (Breeuwer et al., 2003). Unstressed cultures (50 μL) were transferred to sterile petri dishes without their lids, which were placed in a constant temperature humidity chamber, and incubated at 25°C with 20.7% air relative humidity (RH) for air-drying. After drying for 1.5 h, the petri dishes were covered and kept at 21°C with 20.7% RH for 1 year. Periodically, the bacterial survival was determined by conventional colony counting.

### Statistical analysis

Mean values and standard deviations were obtained from three replicate experiments with duplicated plating (*n* = 6). Statistical analysis was performed by Analysis of Variance (ANOVA) with the SPSS 20.0 software. Tukey's multiple range test was used to determine the significant differences (*p* < 0.05) between treatments.

## Results

### Antimicrobial susceptibility tests

The antimicrobial susceptibility pattern and Minimal Inhibitory Concentration (MIC) of the 70 *Cronobacter* strains are shown in Tables 1, 2. All *Cronobacter* strains were susceptible to most antibiotics, including amikacin, ampicillin-sulbactam, aztreonam, cefepime, cefotaxime, ceftazidime, chloramphenicol, ciprofloxacin, colistin, gentamicin, imipenem, levofloxacin, meropenem, moxifloxacin, piperacillin, piperacillin- tazobactam, tetracycline, and trimethoprim-sulfamethoxazole. However, they were resistant to amoxicillin-clavulanate, ampicillin, and cefazolin. The MIC of antibiotics, except cefazolin, did not vary across the 70 *Cronobacter* strains. Ciprofloxacin and trimethoprim-sulfamethoxazole were considered to be the most effective antibiotics against the 70 *Cronobacter* strains at MIC of ≤ 0.5 and ≤ 0.5/0.95 μg/mL. In contrast, there were some differences in their resistance to cefazolin; Table 2. The 70 *Cronobacter* strains were divided into four groups according to their cefazolin MIC-values (≤4, >4, ≤8, >8, ≤16, >16 μg/mL). A majority of *Cronobacter* isolates with the same ST had a same MIC-value.

**Table 1:** **Antimicrobial susceptibility and MIC of 70 ***Cronobacter*** strains isolated from PIF and processing environments**.

**Antibiotics**	**MIC^a^ (μg/mL)**	**SIR^b^**	**Antibiotics**	**MIC^a^ (μg/mL)**	**SIR^b^**
Amikacin	≤8	S (100%)	Colistin	≤0.5	S (100%)
Amoxicillin-Clavulanate	≤4/2	R (100%)	Gentamicin	≤2	S (100%)
Ampicillin	≤4	R (100%)	Imipenem	≤1	S (100%)
Ampicillin-Sulbactam	≤4/2	S (100%)	Levofloxacin	≤1	S (100%)
Aztreonam	≤2	S (100%)	Meropenem	≤1	S (100%)
Cefotaxime	≤1	S (100%)	Moxifloxacin	≤1	S (100%)
Ceftazidime	≤1	S (100%)	Piperacillin	≤4	S (100%)
Cefepime	≤2	S (100%)	Piperacillin-tazobactam	≤4/4	S (100%)
Chloramphenicol	≤4	S (100%)	Tetracycline	≤2	S (100%)
Ciprofloxacin	≤0.5	S (100%)	Trimethoprim-sulfamethoxazole	≤0.5/0.95	S (100%)

a *MIC: (Minimal Inhibitory Concentration) the lowest concentration of the antibiotics that can inhibit effectively growth of the tested microorganism*.

b *S, Susceptible; I, Intermediate; R, Resistant*.

**Table 2:** **The differences of resistance to cefazolin in 70 ***Cronobacter*** strains isolated from PIF and processing environments**.

**MLST**	**Stain**	**MIC-1**	**MIC-2**	**MIC-3**	**MIC-4**	**MLST**	**Stain**	**MIC-1**	**MIC-2**	**MIC-3**	**MIC-4**
ST1	CE21					ST12	CE41				
	CE24						CE44				
	CE43					ST17	CE28				
	CE47						CE58				
	CE59					ST21	CE51				
	CE60						CE52				
	CE63						CE53				
	CE69					ST22	CE15				
	CE70						CE50				
	CE71					ST31	CE56				
	CE72					ST40	CE29				
	CE73					ST50	CE32				
	CE74					ST64	CE25				
	CE79						CE30				
ST4	CE1						CE31				
	CE7						CE33				
	CE9						CE34				
	CE10						CE36				
	CE11						CE54				
	CE12						CE62				
	CE17						CE68				
	CE18						CE77				
	CE19						CE78				
	CE20					ST83	CE8				
	CE22					ST201	CM35				
	CE23					ST258	CM2				
	CE27						CM3				
	CE48						CM5				
	CE49					ST259	CE16				
	CE61					ST268	CE26				
	CE64					ST260	CE55				
	CE67					ST261	CE75				
ST8	CE13						CE76				
	CE14					ST269	CE65				
ST12	CE38						CE66				

**Table:** 

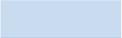	*MIC-1*	*(≤4 μg/mL)*
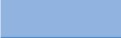	*MIC-2*	*(> 4, ≤8 μg/mL)*
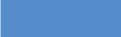	*MIC-3*	*(> 8, ≤16 μg/mL)*
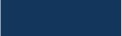	*MIC-4*	*(> 16, μg/mL)*.

### Resistance to osmotic stress

Six *C. sakazakii* and two *C. malonaticus* strains were incubated in BHI with various molar equivalents of either NaCl or sorbitol at 37°C for 24 h. The TTD values and growth rates of eight *Cronobacter* strains are shown in Table 3. Compared with growth in BHI without high concentration solutes, the growth of all eight strains in BHI with different concentrations of NaCl and sorbitol was significantly delayed and slower. The TTD-values of all eight strains growing at >8% NaCl were >24 h, and the growth rates of those ranged from 0.0004/h to 0.0035/h. Meanwhile, in BHI with sorbitol, all strains reached an OD 600 of 0.2 within 12 h. This result showed that the growth of strains was not completely inhibited in <31% sorbitol, whereas there was no growth in the molar equivalent NaCl. In addition, there was variation between strains in their resistance to osmotic stress. Among the eight strains in this study, the growth of CE25 (ST64) was the most significantly delayed (*p* < 0.05), while CE13 (ST8) had more resistance to the delayed growth compared with other strains. The growth rates of CE13 (ST8) and CE1 (ST4) were significantly higher (*p* < 0.05) than that of other strains, and CM35 (ST201) was the slowest grower (*p* < 0.05) in all strains.

**Table 3:** **Growth ability of the six ***C. sakazakii*** (CE21, CE1, CE13, CE38, CE52, and CE25) and two ***C. malonaticus*** (CM35 and CM3) strains under concentrations of NaCl (4, 6, 8, 10% w/v) and sorbitol (SB) (12.5, 19, 25, 31%w/v) conditions**.

	**Solute (w/v)**	***Cronobacter* strains**							
		***C. sakazakii***						***C. malonaticus***	
		**CE21 (ST1)**	**CE1 (ST4)**	**CE13 (ST8)**	**CE38 (ST12)**	**CE52 (ST21)**	**CE25 (ST64)**	**CM35 (ST201)**	**CM3 (ST258)**
TTD (h)	Control (0%)	3	3	3	3	3	3	3	3
	NaCl (4%)	7	5	5	7	5	9	9	6
	NaCl (6%)	10	6	6	12	8	17	14	11
	NaCl (8%)	>24	>24	>24	>24	>24	>24	>24	>24
	NaCl (10%)	>24	>24	>24	>24	>24	>24	>24	>24
	SB (12.5%)	5	3	4	5	4	5	5	5
	SB (19%)	7	5	5	6	6	7	7	7
	SB (25%)	8	7	7	7	7	8	9	9
	SB (31%)	11	9	7	9	9	12	12	12
Growth Rate	Control (0%)	0.064 ± 0.001^a^	0.065 ± 0.003^a^	0.064 ± 0.002^a^	0.065 ± 0.002^a^	0.064 ± 0.002^a^	0.065 ± 0.002^a^	0.065 ± 0.002^a^	0.065 ± 0.001^a^
	NaCl (4%)	0.029 ± 0.003^adef^	0.036 ± 0.002^bce^	0.038 ± 0.003^bc^	0.032 ± 0.004^ade^	0.036 ± 0.002^bce^	0.034 ± 0.002^abde^	0.029 ± 0.003^adef^	0.026 ± 0.002^af^
	NaCl (6%)	0.017 ± 0.002^a^	0.030 ± 0.002^b^	0.026 ± 0.001^c^	0.020 ± 0.002^d^	0.024 ± 0.002^c^	0.018 ± 0.001^a^	0.016 ± 0.002^ae^	0.015 ± 0.002^e^
	NaCl (8%)	0.002 ± 0.000^a^	0.004 ± 0.000^b^	0.003 ± 0.000^c^	0.001 ± 0.000^d^	0.001 ± 0.000^d^	0.001 ± 0.000^d^	0.001 ± 0.000^d^	0.001 ± 0.000^d^
	NaCl (10%)	0.001 ± 0.000^a^	0.001 ± 0.000^a^	0.001 ± 0.000^a^	0.001 ± 0.000^a^	0.001 ± 0.000^a^	0.000 ± 0.000^b^	0.0004 ± 0.000^b^	0.000 ± 0.000^b^
	SB (12.5%)	0.053 ± 0.002^a^	0.059 ± 0.001^b^	0.058 ± 0.002^b^	0.060 ± 0.001^b^	0.060 ± 0.001^b^	0.059 ± 0.001^b^	0.048 ± 0.002^c^	0.046 ± 0.002^c^
	SB (19%)	0.047 ± 0.001^a^	0.051 ± 0.002^bcd^	0.053 ± 0.002^bc^	0.051 ± 0.001^bcd^	0.052 ± 0.001^bcd^	0.050 ± 0.002^bd^	0.039 ± 0.001^e^	0.037 ± 0.001^e^
	SB (25%)	0.040 ± 0.001^a^	0.043 ± 0.001^b^	0.045 ± 0.001^c^	0.038 ± 0.001^a^	0.039 ± 0.001^a^	0.039 ± 0.001^a^	0.030 ± 0.001^d^	0.030 ± 0.001^d^
	SB (31%)	0.024 ± 0.001^aeh^	0.035 ± 0.001^b^	0.038 ± 0.001^c^	0.026 ± 0.001^de^	0.025 ± 0.001^aeh^	0.028 ± 0.000^f^	0.022 ± 0.000^g^	0.025 ± 0.001^aeh^

*All strains have different STs. The averages of three replications ± standard deviations were given. Rows values with the different letters were significant different (p < 0.05); The p value for each comparison is showed on each row*.

### Resistance to dry stress

The survival of six *C. sakazakii* and two *C. malonaticus* strains stored under desiccation condition (21°C, RH = 20.7%) was monitored for up to 1 year (Table 4). In the first 60 days, the average recovery declined by about 1.55 log cycles, then, a smaller decrease was observed during the next 10 months, ranging from 1.07 log cycles (CE13, ST8) to 1.85 log cycles (CE25, ST64). Within 1 year, the reduction of all strains ranged from 2.43 log cycles (CE13, ST8) to 3.46 log cycles (CE25, ST64). The survival of *C. malonaticus* CM3 (ST258) and *C. sakazakii* CE25 (ST64) were significantly less (*p* < 0.05) than the other six *Cronobacter* strains, and *C. sakazakii* CE13(ST8) was the highest (*p* < 0.05) survival in all eight *Cronobacter* strains. In addition, during the different time-phase in 1 year, the CM3 (ST258) had significant lower (*p* < 0.05) survival value compared with other strains.

**Table 4:** **Survivors (log cfu/mL) of the six ***C. sakazakii*** (CE21, CE1, CE13, CE38, CE52, and CE25) and two ***C. malonaticus*** (CM35 and CM3) after dehydration for up to 1 year**.

**Time (days)**	**Survivors (log cfu/mL)**							
	***C. sakazakii***						***C. malonaticus***	
	**CE21 (ST1)**	**CE1 (ST4)**	**CE13(ST8)**	**CE38 (ST12)**	**CE52 (ST21)**	**CE25 (ST64)**	**CM35 (ST201)**	**CM3 (ST258)**
0	8.00	8.00	8.00	8.00	8.00	8.00	8.00	8.00
6	7.81 ± 0.15^a^	7.86 ± 0.11^a^	7.88 ± 0.02^a^	7.89 ± 0.03^a^	7.81 ± 0.06^a^	7.65 ± 0.03^b^	7.77 ± 0.01^a^	7.48 ± 0.05^c^
18	7.62 ± 0.04^ad^	7.51 ± 0.04^b^	7.70 ± 0.04^cd^	7.81 ± 0.03^d^	7.67 ± 0.04^acd^	7.28 ± 0.04^e^	7.53 ± 0.03^b^	7.05 ± 0.05^g^
45	6.64 ± 0.16^a^	7.10 ± 0.05^b^	7.03 ± 0.05^b^	7.14 ± 0.05^b^	7.00 ± 0.04^b^	6.65 ± 0.10^a^	6.71 ± 0.06^a^	6.24 ± 0.05^c^
60	6.45 ± 0.10^abde^	6.58 ± 0.10^abcd^	6.60 ± 0.10^abcd^	6.62 ± 0.08^bcd^	6.51 ± 0.07^acde^	6.39 ± 0.09^ade^	6.48 ± 0.13^abcde^	6.07 ± 0.11^f^
90	6.29 ± 0.10^abef^	6.47 ± 0.09^abcdf^	6.51 ± 0.12^bcdf^	6.50 ± 0.07^bcdf^	6.38 ± 0.11^abdf^	6.18 ± 0.10^aef^	6.33 ± 0.08^abcdef^	5.92 ± 0.16^g^
120	6.11 ± 0.10^ad^	6.28 ± 0.06^bcd^	6.40 ± 0.07^bc^	6.30 ± 0.09^bcd^	6.11 ± 0.12^ad^	6.07 ± 0.09^ad^	6.21 ± 0.09^abd^	5.81 ± 0.09^e^
165	5.86 ± 0.10^abe^	5.95 ± 0.11^abd^	6.29 ± 0.13^c^	6.11 ± 0.09^bd^	5.85 ± 0.10^abe^	5.77 ± 0.13^ae^	5.89 ± 0.06^abe^	5.31 ± 0.05^f^
225	5.48 ± 0.12^a^	5.38 ± 0.10^a^	6.02 ± 0.10^b^	5.85 ± 0.08^c^	5.35 ± 0.11^a^	5.38 ± 0.08^a^	5.49 ± 0.06^a^	5.02 ± 0.05^d^
255	5.27 ± 0.13^acd^	5.29 ± 0.11^acd^	5.86 ± 0.10^b^	5.72 ± 0.07^b^	5.16 ± 0.08^ac^	5.16 ± 0.06^ac^	5.38 ± 0.11^ad^	4.95 ± 0.09^e^
365	4.96 ± 0.12^a^	5.00 ± 0.08^a^	5.57 ± 0.10^b^	5.39 ± 0.10^c^	4.86 ± 0.09^a^	4.54 ± 0.09^d^	4.97 ± 010^a^	4.56 ± 0.11^d^
Total decrease	3.04 ± 12^a^	3.00 ± 0.08^a^	2.43 ± 0.10^b^	2.61 ± 0.10^c^	3.14 ± 0.09^a^	3.46 ± 0.09^d^	3.03 ± 010^a^	3.44 ± 0.11^d^

*The averages of three replications ± standard deviations are shown. All strains have different STs. Rows values with the different letters were significant different (p < 0.05). The p value for each comparison is showed on each row*.

## Discussion

*C. sakazakii* and *C. malonaticus* are the dominant species of *Cronobacter* spp. isolated from PIF and processing environment, and can infect infants and adults, respectively (Fei et al., 2015). Given the long term shelf-life of PIF (up to 1 year) and repeated isolation from PIF production plants (Craven et al., 2010; Sonbol et al., 2013), environmental fitness of *Cronobacter* is very important trait to understand as it may lead to increased persistence and neonatal exposure.

The phylogenetic relationship of concatenated sequences (3,036 bp) based on conventional MLST (7 loci), ribosomal-MLST (53 loci), and core genome MLST (1865 loci) reflects the whole genome phylogeny of the *Cronobacter* genus (Joseph et al., 2012b; Forsythe et al., 2014). The application of MLST has also led to the recognition of stable clonal complexes and sequence types associated with certain clinical presentations. Subsequently the diversity and clonal stability within the *Cronobacter* genus needs to be taken into consideration when interpreting laboratory studies. Therefore, *Cronobacter* strains with different sequence types were chosen for detailed study here.

In this study, 70 *Cronobacter* strains, previously isolated from PIF and processing environments, were used. These represented 19 sequence types of *C. sakazakii* and *C. malonaticus*, detailed information can be obtained from *Cronobacter* MLST databases (http://pubmlst.org/cronobacter/) (Fei et al., 2015). The main STs from PIF and processing environments, were the neonatal pathovars *C. sakazakii* ST4, ST1, and ST8, as well as ST64, ST12, ST21, ST258, and ST201.

The isolates were susceptible to most antibiotics, except for amoxicillin-clavulanate, and ampicillin. Previous reports revealed that the occurrence of susceptibility to cefotaxime, chloramphenicol, ciprofloxacin, gentamicin, and tetracycline in *Cronobacter* strains isolated from different sources (Chon et al., 2012; Xu et al., 2014). Chon et al. (2012) reported that only 5.6% strains from desiccated foods in Korea were resistance to ampicillin whereas in our study, all isolates were resistant to this antibiotic. Furthermore, we found that the MIC values of cefazolin varied across different STs, but were similar within each ST. This may reflect a connection between the ST and antimicrobial resistance.

The ability of eight *Cronobacter* isolates with different STs to resist the osmotic stress was evaluated in this study. Compared with *C. malonaticus* isolates, *C. sakazakii* isolates had a higher capacity to grow in the hyperosmotic media, in agreement with previous studies (Caubilla-Barron et al., 2007; Avelino Alvarez-Ordóñez et al., 2014). *C. sakazakii* CE13 (ST8) was the most resistant to osmotic stress in the eight strains, interestingly, *C. sakazakii* ATCC 29544^T^ reported to have atypical growth characteristics is also ST8 (Osaili and Forsythe, 2009). *C. sakazakii* CE1 (ST4) also showed a more osmotic stress resistance, which might be one of the major reasons why *C. sakazakii* CC4 is frequently isolated from infant food, ready-to-eat foods, potable water. This could result in higher infant exposure and therefore risk of infection (Forsythe et al., 2014).

*Cronobacter* spp. tend to persist more in PIF with a water activity (*a*_w_) 0.25–0.30, compared with *a*_w_ 0.43–0.50, and the survival rate was not associated with whether the PIF was milk-based or soybean-based (Osaili and Forsythe, 2009). In general, this organism can persist in PIF for more than 1 year, therefore, monitoring of survival under desiccated conditions is warranted for the two main *Cronobacter* species, *C. sakazakii* and *C. malonaticus*. Breeuwer et al. (2003) reported that the reduction of *Cronobacter* strains (species undetermined) which had been dried in air and incubated for 46 days at 25°C ranged from 1.0 to 1.5 log cycles. Using similar desiccation conditions, the viable counts of *Cronobacter* strains maintained at 25°C for 45 days decreased by 0.86–1.76 log cycles in our study. Barron and Forsythe (2007) reported that the number of survivors of *C. sakazakii, C. muytjensis*, and *C. turicensis* under desiccated conditions in the first 30 days reduced by 0.58 log cycles on average, and decreased 3.34 log cycles during the first 6 months. While, under the similar desiccation conditions, the results in current study indicated that the reduction of *Cronobacter* strains declined by an average of 0.86 log cycles during the first month, and 2.25 log cycles during the first 6 months. The differences in reduction of *Cronobacter* strains between different reports indicated that a continuous assessment of resistance to dry stress was very necessary.

Interestingly, after kept in the desiccation conditions for 1 year, the total decrease of CE13 (ST8) was the least, followed by CE38 (ST12) and CE1 (ST4). This trend was similar to osmotic stress resistance. Mechanisms of resistance to these environmental stressor have previously been reviewed by Osaili and Forsythe (2009). The higher resistance to both osmotic and desiccation stresses by *C. sakazakii* compared with *C. malonaticus* may in part account for the predominance of *C. sakazakii* in PIF and processing environments and subsequent greater infant exposure.

In summary, this study contributes to an improved understanding of the environmental persistence of *C. sakazakii* and *C. malonaticus* and subsequent risk of infant exposure through contaminated PIF. In addition, the relatively low antibiotic resistance is reassuring for the current treatment of *Cronobacter* infections.

## Author contributions

Conceived and designed the experiments: CM and PF. Performed the experiments: PF, YJ, CM, JF, and YZ. Generated and analyzed the data: PF and RL. Wrote the paper: PF, CM, and SF.

### Conflict of interest statement

The authors declare that the research was conducted in the absence of any commercial or financial relationships that could be construed as a potential conflict of interest.
